# Omicron COVID-19 Case Estimates Based on Previous SARS-CoV-2 Wastewater Load, Regional Municipality of Peel, Ontario, Canada

**DOI:** 10.3201/eid2908.221580

**Published:** 2023-08

**Authors:** Lydia Cheng, Hadi A. Dhiyebi, Monali Varia, Kyle Atanas, Nivetha Srikanthan, Samina Hayat, Heather Ikert, Meghan Fuzzen, Carly Sing-Judge, Yash Badlani, Eli Zeeb, Leslie M. Bragg, Robert Delatolla, John P. Giesy, Elaine Gilliland, Mark R. Servos

**Affiliations:** Regional Municipality of Peel, Mississauga, Ontario, Canada (L. Cheng, M. Varia, K. Atanas, E. Gilliland);; University of Waterloo, Waterloo, Ontario, Canada (H.A. Dhiyebi, N. Srikanthan, S. Hayat, H. Ikert, M. Fuzzen, C. Sing-Judge, Y. Badlani, E. Zeeb, L.M. Bragg, M.R. Servos);; University of Ottawa, Ottawa, Ontario, Canada (R. Delatolla);; University of Saskatchewan, Saskatoon, Saskatchewan, Canada (J.P. Giesy);; Baylor University, Waco, Texas, USA (J.P. Giesy)

**Keywords:** COVID-19, 2019 novel coronavirus disease, coronavirus disease, severe acute respiratory syndrome coronavirus 2, SARS-CoV-2, viruses, respiratory infections, zoonoses, wastewater-based epidemiologic monitoring, public health surveillance, Regional Municipality of Peel, Canada

## Abstract

We determined correlations between SARS-CoV-2 load in untreated water and COVID-19 cases and patient hospitalizations before the Omicron variant (September 2020–November 2021) at 2 wastewater treatment plants in the Regional Municipality of Peel, Ontario, Canada. Using pre-Omicron correlations, we estimated incident COVID-19 cases during Omicron outbreaks (November 2021–June 2022). The strongest correlation between wastewater SARS-CoV-2 load and COVID-19 cases occurred 1 day after sampling (r = 0.911). The strongest correlation between wastewater load and COVID-19 patient hospitalizations occurred 4 days after sampling (r = 0.819). At the peak of the Omicron BA.2 outbreak in April 2022, reported COVID-19 cases were underestimated 19-fold because of changes in clinical testing. Wastewater data provided information for local decision-making and are a useful component of COVID-19 surveillance systems.

Public health surveillance of COVID-19 activity has expanded from monitoring of persons with laboratory-confirmed SARS-CoV-2 infection to including wastewater surveillance. Studies conducted in early 2020 provided proof of concept that wastewater surveillance of SARS-CoV-2 can be used to determine prevalence of COVID-19 in several countries (*1*–*3*). Other studies have since shown the viability of that surveillance indicator (*4*–*7*) with varying success when biomarkers such as pepper mild mottle virus (PMMoV) and crAssphage were used to normalize the measurements of SARS-CoV-2 in fecal matter in samples (*4*,*8*,*9*). Wastewater surveillance indicators become especially relevant when PCR testing eligibility changed or when clinical testing capacity was overwhelmed, resulting in an incomplete picture of local COVID-19 activity.

The Regional Municipality of Peel in Ontario, Canada (hereafter referred to as Peel) serves 1.5 million residents of the cities/towns of Brampton, Caledon, and Mississauga in Ontario, Canada (*10*). As of July 16, 2022, the COVID-19 incidence rate in Peel was one of the highest in Ontario; cumulative incidence was 12,098 laboratory-confirmed COVID-19 cases per 100,000 Peel residents, compared with 9,164/100,000 Ontario residents (*11*). Since April 2020, Peel has sampled untreated wastewater from its 2 wastewater treatment plants (WWTPs) and tested it for SARS-CoV-2. Peel’s WWTPs, serving ≈96% of the region’s residential postal codes, are Clarkson (population served 643,331) and G.E. Booth (population served 1,089,738).

On December 30, 2021, as the Omicron BA.1 variant surged in Ontario, the province restricted clinical PCR testing (which had previously been available to any symptomatic person or close contact of a COVID-19 case-patient) to groups at greatest risk, including hospitalized patients, patient-facing healthcare workers, and staff and residents in hospitals and congregate living settings (*12*). This policy change, the only one to substantially affect the number of completed tests during our study period, resulted in a shift in Peel’s COVID-19 surveillance strategy. Wastewater surveillance of SARS-CoV-2 became a requisite tool for monitoring community-level COVID-19 activity and was used as a key indicator to provide information for local public health decision-making and communication (*13*).

We report correlations between clinical COVID-19 indicators (reported cases and hospitalizations) and SARS-CoV-2 load in untreated wastewater at various lags across COVID-19 pandemic waves 2–6 in Peel during August 2020–June 2022. We also estimated the number of incident COVID-19 cases in Peel, on the basis of SARS-CoV-2 load in wastewater before the clinical PCR testing policy change, during the Omicron outbreaks when PCR testing was restricted. Last, we assessed the usefulness of normalizing SARS-CoV-2 concentrations to PMMoV.

## Methods

### Wastewater Sampling and SARS-CoV-2 RNA Measurement

We sampled wastewater from G.E. Booth and Clarkson WWTPs, located in Mississauga, 3–5 weekdays per week, according to the needs of Peel Public Health and the recommendations of the US Centers for Disease Control and Prevention (*14*) (Appendix Table 1). We obtained daily flow rates (m^3^/day) for each WWTP. In total, we included 356 samples from G.E. Booth and 359 samples from Clarkson in this study.

We collected 24-hour composite samples of untreated wastewater influent, before any screening or grit removal, by using Hach model AS950 automatic samplers (https://www.hach.com) and stored them in high-density polyethylene containers. We kept containers at 4°C and transported them to the University of Waterloo (Waterloo, ON, Canada) for extraction and quantification of SARS-CoV-2 RNA. Sample analysis and reporting occurred within 2 weeks of collection; most samples were analyzed within the same week.

We used a polyethylene glycol precipitation method (*2*) for each wastewater sample as follows: we added 40 mL of sample to a 50-mL centrifuge tube with polyethylene glycol (4 g) and NaCl (0.9 g) and spiked a surrogate (e.g., human coronavirus 229E or murine hepatitis virus) into the sample. The sample was shaken on ice for 2 h and left to settle at 4°C overnight. We then centrifuged the sample at 12,000 × *g* for 1.5 h to concentrate the virus into the solids with the supernatant discarded. We extracted SARS-CoV-2 RNA and purified it from the solids by using either TRIzol reagent (Invitrogen, https://www.thermofisher.com) or Power Microbiome Kit (QIAGEN, https://www.qiagen.com), following the manufacturer’s protocol, with up to 250 mg (wet weight) of the pellet resuspended in either TriZOL or TriZOL/PM1 solution. We eluted the RNA in 100 μL nuclease-free water. Extracted RNA then underwent 1-step quantitative reverse transcription PCR for SARS-CoV-2 (N1, N2 gene targets [*15*]) and PMMoV (*16*) (Appendix).

### COVID-19 Case-Patient, Hospitalization, and Testing Data

We extracted nonnominal data for patients who met the provincial case definition of having confirmed or probable COVID-19 (*17*). Data fields included residential postal code and episode date (earliest date of symptom onset, specimen collection, or date reported). Case-patient data were restricted to persons who had a permanent residential address in Peel at the time of COVID-19 diagnosis and who experienced episodes from August 30, 2020, through June 18, 2022 (n = 185,895). We classified case-patients by sewershed—G.E. Booth, Clarkson, septic system, or unknown—on the basis of residential postal code matched to the 2019 Postal Code Conversion File (https://www.canadapost-postescanada.ca/cpc/doc/en/marketing/postal-code-conversion-file-reference-guide.pdf), which we spatially joined with the Peel sewershed geographic boundary file. We validated postal codes that did not match with the Postal Code Conversion File by using the Canada Post Find a Postal Code web tool (https://www.canadapost-postescanada.ca/info/mc/personal/postalcode/fpc.jsf). We then aggregated case-patients by episode date and sewershed. Among 185,895 COVID-19 case-patients, 1.5% were not matched to either WWTP sewershed, 0.9% were associated with septic systems, and 0.6% were unable to be matched to the geographic boundary file.

Patient hospitalization information, obtained from COVID-19 case follow-up, is underreported in the Ontario Ministry of Health’s COVID-19 case registry, Case and Contact Management Solution. Therefore, we acquired aggregate COVID-19 patient hospitalization data from the Ontario Ministry of Health’s Daily Bed Census, for August 30, 2020, through June 18, 2022 (https://data.ontario.ca/dataset/bed-census-summary-bcs). We extracted the daily number of acute care admissions among laboratory-confirmed COVID-19 patients, regardless of patient residence or reason for admission, to the 3 acute hospitals located in Peel (Trillium Health Partners [Credit Valley Hospital and Mississauga Hospital] and William Osler Health System [Brampton Civic Hospital]). To describe SARS-CoV-2 clinical testing trends during the study period, we extracted completed PCR tests for Peel residents from the Ontario Ministry of Health Ontario Laboratory Information System, by week of specimen collection.

### Data Processing

We categorized data from August 30, 2020, through June 18, 2022, by epidemic wave, each characterized by the dominance of the wild-type or a variant of SARS-CoV-2. We reported SARS-CoV-2 N-gene values as the mean concentration (copies/mL) of the N1 and N2 gene targets for each sampling date and WWTP. We retained in the dataset mean concentration values below the limit of detection (0.5 copies/mL) or limit of quantification (3.5 copies/mL) as reported from the quantitative reverse transcription PCR analysis. Normalized data were presented as the mean concentration of N1 and N2 divided by the concentration of PMMoV. We calculated daily load per WWTP by multiplying the flow rate by the mean N-gene concentration or PMMoV-normalized data.

To visualize trends at each sewershed, we plotted daily wastewater loads and COVID-19 cases. We assessed wastewater load and case data for normality by visually inspecting quantile-quantile plots and histograms after applying various data transformations. The natural log transformation, after adding a constant of 1 (ln[x + 1]), resulted in approximately normally distributed data in both datasets (Appendix Figure 1). To assess the strength of linear associations between log-transformed SARS-CoV-2 loads in wastewater and log-transformed COVID-19 cases, we computed Pearson correlation coefficients (*r*) by using lags of 0–5 days between sampling date and case episode date. We repeated that analysis by using PMMoV-normalized, log-transformed wastewater load data. We considered correlation coefficients to be significant at p<0.05.

Because the number of COVID-19 patient hospitalizations at Peel hospitals were not specific to sewersheds, we summed the daily wastewater loads at both WWTPs, applied the ln(x + 1) transformation because it resulted in more normally distributed data, and calculated r between log-transformed total N-gene load and log-transformed hospitalizations. We computed correlation coefficients per wave at lags of 1–14 days between wastewater sampling date and hospitalization date. We repeated that analysis by using log-transformed PMMoV-normalized wastewater data.

### Wastewater-to-Case Ratios, Linear Regression, and Case Estimations

After observing the strongest linear association between SARS-CoV-2 load in wastewater and reported COVID-19 cases at a 1-day lag, we computed the median daily wastewater-to-case ratio per wave. We compared these median wastewater-to-case ratios per wave by using the Kruskal-Wallis test, followed by the Dunn test for pairwise comparisons (*18*). Because clinical PCR testing eligibility was limited as of December 30, 2021, we hypothesized that laboratory-confirmed COVID-19 cases during the Omicron waves were underestimated. We therefore used wastewater load and reported case data in the pre-Omicron waves to fit a model to estimate reported case incidence during Omicron waves. We created a simple linear regression model by using the sum of the daily transformed N-gene load in wastewater at the 2 WWTPs and daily transformed COVID-19 case counts at the 2 WWTPs at a 1-day lag during the pre-Omicron waves (2–4), and we estimated the number of cases, with corresponding 95% prediction intervals, for the Omicron waves (5 and 6), on the basis of the measured wastewater SARS-CoV-2 load. For statistical analyses, we used Stata version MP17.0 (StataCorp LLC, https://www.stata.com).

## Results

### Trends in Wastewater SARS-CoV-2 Load and COVID-19 Clinical Indicators

**Figure 1:**
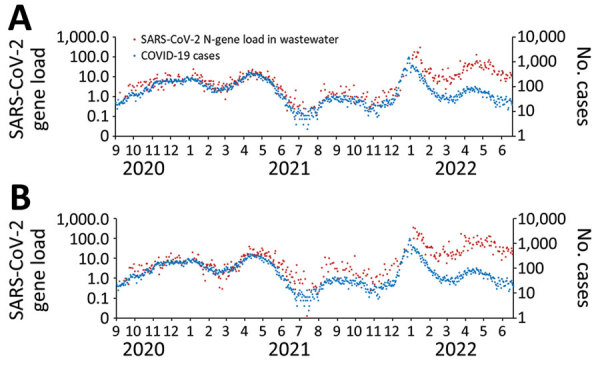
Mean SARS-CoV-2 N-gene load (10^12^ copies/d) in untreated wastewater at Clarkson Wastewater Treatment Plant and reported COVID-19 case-patients residing in the Clarkson sewershed, Regional Municipality of Peel, Ontario, Canada, September 1, 2020–June 18, 2022. A) Nonnormalized; B) pepper mild mottle virus normalized. Data are plotted on the logarithmic scale.

**Figure 2:**
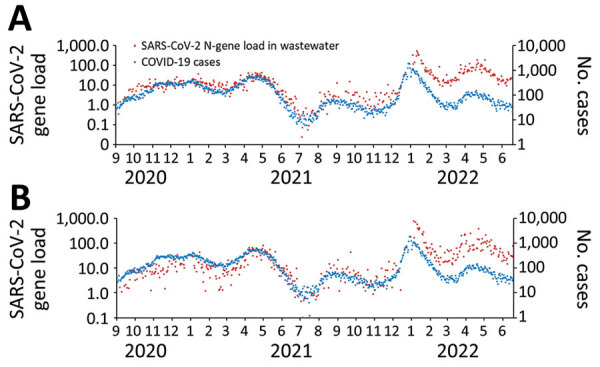
Mean SARS-CoV-2 N-gene load (10^12^ copies/d) in untreated wastewater at G.E. Booth Wastewater Treatment Plant and reported COVID-19 case-patients residing in the G.E. Booth sewershed, Regional Municipality of Peel, Ontario, Canada, September 1, 2020–June 18, 2022. A) Nonnormalized; B) pepper mild mottle virus normalized. Data are plotted on the logarithmic scale.

**Figure 3:**
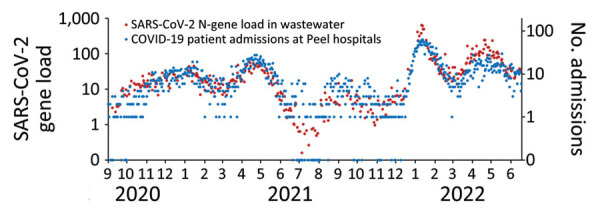
Mean combined SARS-CoV-2 N-gene loads (10^12^ copies/d) in untreated wastewater at Clarkson and G.E. Booth Wastewater Treatment Plants and acute-care admissions of confirmed COVID-19 patients at Peel hospitals, Regional Municipality of Peel, Ontario, Canada, September 1, 2020–June 18, 2022. Data are plotted on the logarithmic scale. For data visualization purposes, daily hospitalization values of zero were converted to 0.1 and are shown along the x-axis.

During waves 2–4 (August 30, 2020–November 28, 2021, before Omicron emerged), the trends and magnitude of reported COVID-19 cases were temporally associated with both the non-normalized SARS-CoV-2 load and PMMoV-normalized load in wastewater (Figures 1, 2). The median daily SARS-CoV-2 load at G.E. Booth was double that of Clarkson, corresponding to the larger size of the G.E. Booth sewershed (Table 1). During waves 2 and 3, the numbers of average weekly clinical PCR tests conducted and overall percent positivity were similar (Table 1; Appendix Figure 1). Furthermore, trends in COVID-19 hospitalizations visually correlated with trends in wastewater load (Figure 3; Appendix Figure 3). During wave 4, dominated by the Delta variant, COVID-19 cases, hospitalizations, and clinical PCR tests were fewer; test positivity was lower; median N-gene loads in wastewater were less; and the proportion of wastewater samples with N-gene concentrations less than the limit of quantification was high (36%).

**Table 1:** Summary of wastewater and COVID-19 clinical data, by wastewater treatment plant and epidemic wave, Regional Municipality of Peel, Ontario, Canada, August 30, 2020–June 18, 2022*

Characteristic	Epidemic wave				
	2, 2020 Aug 30–2021 Feb 20	3, 2021 Feb 21–2021 Jul 17	4, 2021 Jul 18–2021 Nov 27	5, 2021 Nov 28–2022 Mar 12	6, 2022 Mar 13–2022 Jun 18
Predominant COVID-19 variant	Original	Alpha	Delta	Omicron BA.1	Omicron BA.2
No. wastewater sample days					
Clarkson WWTP	89	94	53	56	67
G.E. Booth WWTP	89	95	50	55	67
Median daily load of SARS-CoV-2 in wastewater, N-gene copies × 10^12^ (range)					
Clarkson WWTP	5.2 (0.5–23.8)	4.4 (<0.01–22.8)	1.0 (0.1–4.3)	10.6 (1.1–299.0)	22.8 (6.0–128.5)
G.E. Booth WWTP	10.1 (1.4–37.3)	9.8 (0.02−48.9)	2.6 (0.3−15.0)	24.1 (1.9–537.6)	42.1 (9.6–186.5)
Median PMMoV-normalized daily load of SARS-CoV-2 in wastewater, N-gene copies × 10^2^ (range)					
Clarkson WWTP	4.6 (0.3–20.4)	5.4 (0.01–39.9)	1.7 (0.2–13.4)	20.4 (3.9–357.3)	39.4 (8.1–173.9)
G.E. Booth WWTP	7.9 (1.1–44.4)	10.4 (0.1–83.9)	4.6 (0.5–43.3)	41.8 (1.3–793.6)	60.1 (14.7–389.6)
No. (%) wastewater samples with concentrations below limit of quantification†					
Clarkson WWTP	1 (1.1)	17 (18.1)‡	19 (35.8)	0 (0.0)	0 (0.0)
G.E. Booth WWTP	1 (1.1)	14 (14.7)§	18 (36.0)	0 (0.0)	0 (0.0)
Median daily no. of reported COVID-19 cases (range)					
Clarkson WWTP	109 (13–246)	100 (2–365)	24 (5–44)	83 (19–1,518)	45 (12–101)
G.E. Booth WWTP	189 (17–421)	165 (4–579)	31 (8–69)	136 (26–1,877)	64 (21–150)
Median daily no. acute care admissions of COVID-19 patients at Peel hospitals (range)	5 (0–19)	6 (0–28)	2 (0–10)	12 (0–63)	12 (3–30)
Mean weekly no. clinical SARS-CoV-2 PCR tests in Peel	21,237	21,050	14,125	18,696	8,513
Clinical PCR tests positive for SARS-CoV-2 in Peel, %	8.3	10.2	2.7	16.7	9.0

*WWTP, wastewater treatment plant. †Limit of quantification: 3.5 SARS-CoV-2 N-gene copies per mL. ‡1 sample had N-gene concentration below the limit of detection (0.5 copies/mL). §2 samples had N-gene concentration below the limit of detection (0.5 copies/mL).

By December 2021, when Omicron BA.1 emerged (wave 5), the magnitude of reported cases no longer aligned with the magnitude of wastewater SARS-CoV-2 load (Figures 1, 2). SARS-CoV-2 load at both WWTPs reached a historic peak in January 2022. Of note, the largest number of daily COVID-19 case-patients ever reported in Peel, for both sewersheds, was on December 29, 2021. COVID-19 patient admissions at Peel hospitals also increased during wave 5; daily median was 12 and maximum was 63 admissions. Clinical PCR tests completed among Peel residents increased sharply in late December 2021 and dropped steeply in January 2022 after the change in PCR testing eligibility (Appendix Figure 1). Test positivity peaked at 31.4% during the week ending January 8, 2022.

COVID-19 wave 6, which occurred in Peel in spring 2022, was driven by Omicron BA.2. Daily median SARS-CoV-2 loads in wastewater were greater than in previous waves; however, loads did not exceed maximum daily values observed during the preceding Omicron BA.1 wave. Median daily COVID-19 patient hospitalizations remained similar to those in the previous wave. Weekly clinical SARS-CoV-2 PCR tests dropped by nearly 60% in wave 6, compared with waves 2–3.

### Association between SARS-CoV-2 Concentrations in Wastewater and Clinical Indicators

For waves 2–6 combined, the strongest correlation between nonnormalized SARS-CoV-2 load in wastewater and reported COVID-19 cases occurred on the same day of sampling (G.E. Booth, *r* = 0.6030; Clarkson, *r* = 0.6273; both WWTPs, *r* = 0.6296; Table 2). Before Omicron, the strongest correlations occurred on the same day as sampling at G.E. Booth (*r* = 0.8698) and 1 day after sampling at Clarkson (*r* = 0.8864) and when load data were combined for both WWTPs (*r* = 0.9106). By wave, the strongest correlations occurred during the Alpha-dominant wave 3. Correlation coefficients were poorer as more time passed between the wastewater sample date and incident cases. PMMoV normalization, compared with no normalization, resulted in weaker correlations for both WWTPs before the Omicron waves and for G.E. Booth during the Omicron waves (Appendix Table 2).

**Table 2:** Pearson correlation coefficients (*r*) between ln(x + 1) transformed daily wastewater SARS-CoV-2 load and ln(x + 1) transformed incident COVID-19 cases, by wastewater treatment plant and epidemic wave, at various lags, Regional Municipality of Peel, Ontario, Canada*

WWTP, lag, d	Epidemic waves							
	2	3	4	5	6	2–6	2–4, pre-Omicron	5–6, Omicron
Clarkson								
0	0.7078	0.8809	0.5747	0.7203	0.6655†	0.6273†	0.8677	0.5469†
1	0.7144†	0.8966	0.6661†	0.7248†	0.5933	0.6220	0.8864†	0.5384
2	0.6935	0.8901	0.5041	0.6607	0.5712	0.5849	0.8705	0.4743
3	0.6678	0.9089†	0.5321	0.6155	0.5480	0.5863	0.8788	0.4497
4	0.6653	0.9037	0.4539	0.5791	0.5468	0.5771	0.8727	0.4236
5	0.6550	0.8906	0.5550	0.5298	0.5762	0.5737	0.8699	0.4023
G.E. Booth								
0	0.7173†	0.8993	0.5774†	0.7922†	0.7572†	0.6030†	0.8698†	0.6828†
1	0.6935	0.9068†	0.5167	0.7601	0.7227	0.5932	0.8696	0.6535
2	0.6714	0.8722	0.4735	0.7076	0.7251	0.5529	0.8452	0.6165
3	0.6466	0.8889	0.4923	0.6767	0.7314	0.5650	0.8559	0.5974
4	0.6575	0.8808	0.4425	0.6347	0.7144	0.5592	0.8492	0.5658
5	0.6499	0.8641	0.4998	0.5953	0.7095	0.5467	0.8417	0.5502
Clarkson and G.E. Booth								
0	0.7647†	0.9422	0.6447†	0.7908†	0.7910†	0.6296†	0.9101	0.6601†
1	0.7431	0.9438†	0.6266	0.7655	0.7357	0.6150	0.9106†	0.6341
2	0.7290	0.9246	0.5295	0.7025	0.7276	0.5774	0.8921	0.5830
3	0.7087	0.9326	0.5505	0.6675	0.7186	0.5820	0.8972	0.5616
4	0.7121	0.9292	0.5051	0.6223	0.7017	0.5756	0.8931	0.5282
5	0.7057	0.9163	0.5706	0.5814	0.7111	0.5678	0.8887	0.5115

*Colors indicate strength of association: gray, weak (*r*<0.4); yellow, moderate (0.4≤*r*<0.7); green, strong (*r*>0.7). WWTP, wastewater treatment plant. †Largest *r* per wave.

Pearson correlation coefficients assessing the relationship between total wastewater SARS-CoV-2 load at the 2 WWTPs and total COVID-19 patient hospitalizations were highest during the Alpha-dominant wave 3 (Table 3; *r* = 0.8679 at a 4-day lag) and the Omicron BA.1-dominant wave 5 (*r* = 0.9161 at a 7-day lag). In the Delta-dominant wave 4, associations were weak and some were not statistically significant. Overall, across the study period, the greatest correlation occurred between wastewater load and hospitalizations 4 days after sampling (*r* = 0.8189). PMMoV normalization resulted in weaker correlation coefficients, and the greatest coefficient occurred 1 day after sampling (*r* = 0.7883; Appendix Table 3).

**Table 3:** Pearson correlation coefficients (*r*) between ln(x + 1) transformed daily SARS-CoV-2 load in wastewater and ln(x + 1) transformed hospitalizations among COVID-19 patients, by epidemic wave, at various lags, Peel region Regional Municipality of Peel, Ontario, Canada*

Lag, d	Epidemic waves					
	2	3	4	5	6	2–6
1	0.6294	0.7920	0.5681†	0.8841	0.4827	0.8110
2	0.5772	0.7617	NS	0.8968	0.5602	0.7967
3	0.6388	0.7608	0.3798	0.8613	0.5009	0.7889
4	0.5600	0.8679†	NS	0.8781	0.5117	0.8189†
5	0.5666	0.7513	NS	0.8618	0.5008	0.7621
6	0.6121	0.8024	NS	0.8913	0.4187	0.8062
7	0.6531†	0.7860	NS	0.9161†	0.5695	0.8120
8	0.6163	0.7625	0.4292	0.8711	0.5767†	0.8009
9	0.6254	0.8036	0.3733	0.8649	0.4673	0.8163
10	0.5249	0.7694	0.3894	0.8498	0.4713	0.7804
11	0.5955	0.8015	0.1440	0.7630	0.4292	0.7853
12	0.5333	0.7351	0.2552	0.7957	0.4124	0.7608
13	0.6369	0.7758	0.2907	0.7420	0.3114	0.7770
14	0.5650	0.7605	0.1717	0.7474	0.3976	0.7525

*Colors indicate strength of association: gray, weak (*r*<0.4); yellow, moderate (0.4≤*r*<0.7); green, strong (*r*>0.7). NS, not significant (p>0.05). †Largest *r* per wave.

### Estimation of COVID-19 Cases in Peel during the Omicron BA.1 and BA.2 Outbreaks

**Figure 4:**
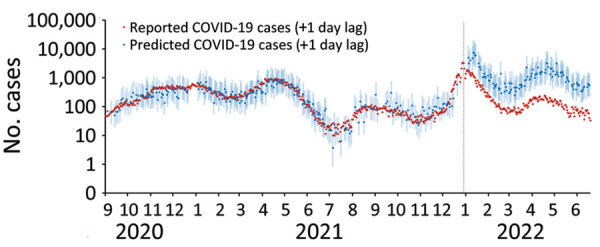
Predicted number of Peel COVID-19 cases (+ 1 day of wastewater sampling date) based on linear regression model using data before wave 5, Regional Municipality of Peel, Ontario, Canada, September 1, 2020–June 18, 2022. Data are plotted on the logarithmic scale. Light blue shaded area represents 95% prediction intervals. Vertical dotted line marks the date when clinical PCR testing for SARS-CoV-2 was restricted to high-risk populations, December 30, 2021.

At each WWTP, the median wastewater-to-case ratios did not statistically differ from each other for waves 2–4. However, the ratios were significantly greater (p<0.05) during waves 5 and 6 (Appendix Table 4, Figure 4). Thus, starting in wave 5, each unit of SARS-CoV-2 load in wastewater was associated with fewer reported COVID-19 cases, as expected, resulting from reduced eligibility for clinical PCR testing.

To estimate the number of COVID-19 cases that would have been reported in waves 5 and 6 had testing eligibility not changed, we created a simple linear regression model by using the summed transformed wastewater load at both WWTPs and summed COVID-19 case data, at a 1-day lag, from waves 2–4 (Figure 4). At the peak measured wastewater load of wave 5 on January 11, 2022, there were 1,160 reported COVID-19 cases, compared with a prediction of 7,515 cases (95% prediction interval 2,871–19,672), representing a 6.5-fold difference. At the peak of wave 6 (greatest measured wastewater load on April 21, 2022), there was an 18.7-fold difference between the estimated number of cases and reported COVID-19 cases (3,170 [95% prediction interval 1,224–8,211] vs. 170 reported cases).

## Discussion

In December 2021, after the emergence of the Omicron variant, to conserve testing capacity the Ontario provincial government implemented changes to PCR test eligibility. As a result, we observed a rapid decrease in completed clinical PCR tests, high percentage positivity, and an increased wastewater-to-case ratio in Peel, indicating an underestimation of reported cases. Similar changes in wastewater-to-case ratios have been reported, resulting from changes in clinical testing strategies in other Canada municipalities (*19*). On the basis of the observed linear association between cases and SARS-CoV-2 load in wastewater in Peel, we estimated that the number of predicted cases was ≈6.5-fold greater than the number of reported cases at the peak of wave 5 (characterized by Omicron BA.1) and ≈18.7-fold greater than reported cases at the peak of wave 6 (Omicron BA.2). In January 2022 (wave 5), Peel Public Health provided near real-time incident case estimations based on wastewater concentrations to Peel hospitals that were experiencing considerable pressures for beds. Those data were, in turn, used to validate the hospitals’ short-term scenario planning and to predict further challenges to hospital occupancy and staffing.

This simple method of case estimation can be easily replicated; however, it relies on linear regression, frequent sampling, and a historical baseline. The linear associations observed in Peel might have been applicable because of sewage collection system characteristics specific to the region. For example, Peel’s storm water and sanitary sewage systems are separated. Furthermore, most of Peel’s wastewater comes from residential sources; during August 2020–June 2022, the source of 72% of wastewater effluent was residential and the sources of 28% were industrial, commercial, or institutional. Last, Peel’s 2 WWTPs serve >95% of Peel’s residents, representing high population-level coverage.

Our study used calculations of load, which require daily measurements of total flow. In earlier analyses, we found similar linear associations between SARS-CoV-2 N-gene concentrations (copies/mL) at the 2 Peel WWTPs and the COVID-19 cases and hospitalizations (data not shown). However, to provide information about Peel as a whole, we found it beneficial to calculate total load to combine data from 2 WWTPs.

Although reported COVID-19 cases during the Omicron waves were known to be an underestimation of true cases, overall trends in reported cases still correlated with trends of SARS-CoV-2 load in wastewater. This association was also observed between SARS-CoV-2 loads in wastewater and hospitalizations of COVID-19 patients during wave 5, which was dominated by Omicron BA.1. This finding might indicate that, within Peel, SARS-CoV-2 load in wastewater might be predictive of hospitalizations for COVID-19, independent of changes in testing uptake, although the optimal lags were variant dependent. The findings of wastewater signal being an indicator of community disease burden were also reported from another Ontario study (*20*). However, because of many complex factors, such as differing virulence of new variants, advancements in treatment, vaccine effectiveness, waning immunity, and outbreaks in hospitals, hospitalizations associated with COVID-19 can vary. Those factors may have explained the poor linear relationship between wastewater concentrations and hospitalizations in the Omicron BA.2–dominant wave 6.

In many wastewater surveillance systems, including the one for Peel, concentrations of SARS-CoV-2 in wastewater are normalized to concentrations of PMMoV, a fecal marker. In our study, PMMoV normalization generally did not improve the correlation between wastewater load and COVID-19 case counts, similar to findings in other studies (*8*,*9*,*21*), or COVID-19 patient hospitalizations, when compared with use of nonnormalized data. This lack of improved correlation might result from the low amount of inflow and infiltration as well as the source of wastewater being mainly residential (72%). Although normalization through PMMoV did not result in improved correlations in Peel, it might be useful in other systems (*4*). Furthermore, monitoring of fecal biomarkers may be used to assess sample quality (e.g., determining whether PMMoV concentrations are within an expected range for a given site).

Our results demonstrate the value of using population-based wastewater surveillance to detect increasing local COVID-19 activity and confirm declining case trends. Among the study limitations, data for numbers of COVID-19 cases included Peel residents and did not include patients who resided in neighboring jurisdictions but who would have contributed to SARS-CoV-2 in the G.E. Booth sewershed. However, we estimated that this contribution was relatively small, considering the large geographic area of the sewershed. Second, the COVID-19 hospitalization data used in our study were aggregated, and we were thus unable to discern whether patients were Peel residents. Third, our application of a historical, pre-Omicron, linear relationship to estimate COVID-19 cases during the Omicron waves was based on the assumption that fecal shedding patterns of SARS-CoV-2 remained the same regardless of SARS-CoV-2 variant, previous infection, and vaccination status. It has been reported that the amount of Omicron BA.2 on nasopharyngeal swabs was double that of Omicron BA.1 (*22*); but we are unaware of recent studies specific to virus in feces. If more fecal virus shedding is produced by Omicron than by other variants, our estimates of case underreporting would be overestimated. Fourth, we are unable to verify the number of true infections without population-level seroprevalence studies. Last, although linear regression is easily understood and accessible, interpretability of our case estimation results is limited as a result of autocorrelation. We found that residuals of the linear regression model exhibited positive autocorrelation, which may have resulted in less precise coefficient estimates and underestimated SEs and 95% prediction intervals. Further work is needed to determine modeling methods that might be more appropriate for analysis of wastewater time-series data (e.g., the SEIR [*23*] and PRESENS [*24*] models) and might also be easily implemented and interpretable by public health authorities. Despite this limitation, there was still value in using a simple method to approximate COVID-19 cases by using wastewater data. Furthermore, our predictions agreed with other estimates projected by hospital partners.

In summary, in the Regional Municipality of Peel, Canada, on the basis of strong historical linear associations between SARS-CoV-2 N-gene load in wastewater and reported COVID-19 cases, we estimated that reported COVID-19 cases were underestimated 19-fold at the peak of the Omicron BA.2 wave in April 2022. As a result of identifying SARS-CoV-2 wastewater load and population-level COVID-19 clinical outcomes, the monitoring of wastewater in Peel has provided critical information about community transmission of COVID-19 that is independent of clinical testing availability and uptake. Wastewater surveillance data can provide information for local decision-making and be a key metric in addition to traditional public health surveillance indicators, particularly for outcomes that are limited by availability of testing information and require triangulation of multiple data sources.

Appendix Additional information for study of Omicron COVID-19 case estimates based on previous SARS-CoV-2 wastewater load, Regional Municipality of Peel, Ontario, Canada.
