# Supplementary material for: Omicron COVID-19 Case Estimates Based on Previous SARS-CoV-2 Wastewater Load, Regional Municipality of Peel, Ontario, Canada
Source: Emerg Infect Dis. 2023 Aug;29(8):1580–8. doi: 10.3201/eid2908.221580 (PMC10370834; doi:10.3201/eid2908.221580)
Supplement: Appendix — Additional information for study of Omicron COVID-19 case estimates based on previous SARS-CoV-2 wastewater load, Regional Municipality of Peel, Ontario, Canada. [file 22-1580-Techapp-s1.pdf]

Article DOI: https://doi.org/10.3201/eid2908.221580

EID cannot ensure accessibility for supplementary materials supplied by authors. Readers who have difficulty accessing supplementary content should contact the authors for assistance.

Omicron COVID-19 Case Estimates Based on Previous SARS-CoV-2 Wastewater Load, Regional Municipality of Peel, Ontario, Canada

Appendix

Methods for Wastewater PCR Quality Assurance and Quality Control

Samples were analyzed with 3 technical replicates and standard curves on each plate. The standard material for the SARS-CoV-2 targets were plasmid (Integrated DNA Technologies (IDT, USA)) or EDX standard (Bio-Rad, USA). G-Blocks (IDT, USA) were used for PMMoV standards. A known RNA standard (MS2, Cedarlane Laboratories, Canada) was added to the qPCR wells to determine if the sample inhibited amplification by qPCR. If inhibition of qPCR was observed, the sample was diluted (1:5 or 1:10) and re-analyzed. Several quality assurance and quality control procedures were conducted with each plate, including: non-template controls, non-reverse transcription control and extraction blanks. Assay efficiency (90%–110%) and R^2 (>0.98) met the MIQE guidelines (*1*).

Reference

1. Bustin SA, Benes V, Garson JA, Hellemans J, Huggett J, Kubista M, et al. The MIQE guidelines: minimum information for publication of quantitative real-time PCR experiments. Clin Chem. 2009;55:611–22. PubMed https://doi.org/10.1373/clinchem.2008.112797

Appendix Table 1. Wastewater treatment plant sampling frequency, September 2020 to June 2022, Peel region

Period	Sampling frequency at Clarkson and G.E. Booth Wastewater Treatment Plants	Rationale
Before September 13, 2020	Once per week (Monday)	Surveillance system was being set up.
September 13, 2020 to October 16, 2020	3 times per week (Monday, Wednesday, Friday)	We increased sampling frequency to 3 per surveillance week.
October 17, 2020 to June 25, 2021	5 times per week (Monday through Friday); 4 if statutory holiday	We increased sampling frequency due to an expected rise in COVID-19 activity in the fall respiratory season.
June 30, 2021 to January 7, 2022	3 times per week (Monday, Wednesday, Friday)	We reduced sampling frequency due to low COVID-19 activity during the Delta-dominant outbreak.
January 10, 2022 to June 18, 2022	5 times per week (Monday through Friday); 4 if statutory holiday	We increased sampling frequency due to the sharp increase in COVID-19 activity due to Omicron BA.1.

Appendix Table 2. Pearson's correlation coefficient (r) between ln(x+1) transformed daily PMMoV-normalized wastewater SARS-CoV-2 load and ln(x+1) transformed incident COVID-19 cases, by wastewater treatment plant and epidemic wave, at various lags, Peel region

WWTP	Lag	Wave 2	Wave 3	Wave 4	Wave 5	Wave 6	Combined Waves 2–6	Combined Waves 2–4 (Pre-Omicron)	Combined Waves 5–6 (Omicron)
Clarkson									
	0 d	0.6958	0.7811	0.5858	0.7507*	0.5518*	0.4926*	0.7439	0.5380*
	1 d	0.7327*	0.7883	0.6901*	0.7438	0.4749	0.4795	0.7572*	0.5153
	2 d	0.7017	0.7836	0.5918	0.6979	0.4075	0.4428	0.7405	0.4574
	3 d	0.6807	0.7924*	0.5467	0.6700	0.3845	0.4431	0.7419	0.4418
	4 d	0.6746	0.7804	0.5131	0.6371	0.4357	0.4329	0.7284	0.4339
	5 d	0.6716	0.7590	0.5719	0.5924	0.4998	0.4312	0.7221	0.4292
G.E. Booth									
	0 d	0.5801*	0.8409*	0.6012*	0.7632*	0.5584	0.4737*	0.7352*	0.6176*
	1 d	0.5699	0.8373	0.5728	0.7488	0.5353	0.4563	0.7189	0.6030
	2 d	0.5550	0.8107	0.5801	0.7150	0.5126	0.4218	0.7044	0.5727
	3 d	0.5123	0.8205	0.4970	0.6855	0.5411	0.4265	0.7014	0.5580
	4 d	0.5286	0.8147	0.4693	0.6564	0.6069*	0.4243	0.6928	0.5527
	5 d	0.5250	0.7918	0.5758	0.6240	0.5918	0.4161	0.6943	0.5414
Clarkson & G.E. Booth									
	0 d	0.6720*	0.8638*	0.6536	0.7976*	0.6358	0.4863*	0.7710*	0.6316*
	1 d	0.6667	0.8518	0.6599*	0.7849	0.5885	0.4630	0.7541	0.6149
	2 d	0.6517	0.8361	0.6246	0.7465	0.5445	0.4286	0.7408	0.5751
	3 d	0.6260	0.8375	0.5552	0.7212	0.5608	0.4303	0.7357	0.5642
	4 d	0.6299	0.8333	0.5614	0.6896	0.5984	0.4263	0.7284	0.5525
	5 d	0.6297	0.8154	0.6525	0.6552	0.6527*	0.4241	0.7311	0.5543

WWTP: Wastewater treatment plant
*Largest r per wave
Note: Cells are colored by strength of association: weak: gray (r<0.4), moderate: yellow (0.4 ≤r <0.7), strong: green (r ≥0.7)

Appendix Table 3. Pearson's correlation coefficient (r) between daily ln(x+1) transformed PMMoV-normalized SARS-CoV-2 N-gene load in wastewater and ln(x+1) transformed hospitalizations among COVID-19 patients, by epidemic wave, at various lags, Peel region

Lag	Wave 2	Wave 3	Wave 4	Wave 5	Wave 6	Combined Waves 2–6
1 d	0.5586	0.7733	0.5959*	0.8310	0.4379	0.7883*
2 d	0.4321	0.7779	0.3871	0.8321	0.5731*	0.7767
3 d	0.4755	0.7547	0.4122	0.8113	0.3300	0.7496
4 d	0.4832	0.8396*	NS	0.8262	0.4362	0.7829
5 d	0.5145	0.7266	NS	0.8132	0.4890	0.7429
6 d	0.5624	0.7588	0.3386	0.8268	0.3467	0.7732
7 d	0.5778*	0.7518	NS	0.8616*	0.5582	0.7778
8 d	0.5612	0.7178	0.4868	0.8492	0.4999	0.7727
9 d	0.5322	0.7683	0.3652	0.8297	0.3860	0.7764
10 d	0.4526	0.7297	0.3948	0.8269	0.3603	0.7445
11 d	0.5525	0.7558	NS	0.7892	0.3166	0.7482
12 d	0.4511	0.6954	NS	0.8071	0.2806	0.7245
13 d	0.5848	0.7210	0.3402	0.7385	0.2566	0.7510
14 d	0.5133	0.7012	NS	0.7991	0.4697	0.7399

NS: Not significant (p ≥ 0.05)
*Largest r per wave
Note: Cells are colored by strength of association: weak: gray (r<0.4), moderate: yellow (0.4 ≤r <0.7), strong: green (r≥0.7)

Appendix Table 4. Ratio of daily SARS-CoV-2 wastewater load to reported Peel COVID-19 cases by wastewater treatment plant and epidemic wave (10^9 N-gene copies/COVID-19 case)

Ratio of wastewater SARS-CoV-2 concentration to COVID-19 cases (10^9 N-gene copies/case)	Wave 2	Wave 3	Wave 4	Wave 5	Wave 6
Clarkson WWTP					
Median	52.5	42.1	38.6	169.1*	486.1*
25th percentile	34.4	30.4	28.2	108.9	323.8
75th percentile	70.7	58.4	69.3	250.4	689.3
Minimum	13.4	0.5	11.7	7.4	154.4
Maximum	124.4	295.7	151.4	1,642.7	1,493.8
G.E. Booth WWTP					
Median	54.1	54.2	64.2	299.0*	552.8*
25th percentile	42.2	38.5	41.2	192.4	445.1
75th percentile	69.4	73.5	96.8	418.2	755.6
Minimum	16.4	1.7	5.4	23.9	175.9
Maximum	172.6	190.3	429.8	1,386.4	1,965.0

*For both treatment plants, the median wave 5 and wave 6 wastewater-to-case ratios were significantly different from each other, and from wave 2, wave 3, and wave 4 ratios ($p < 0.001$ using Dunn test)

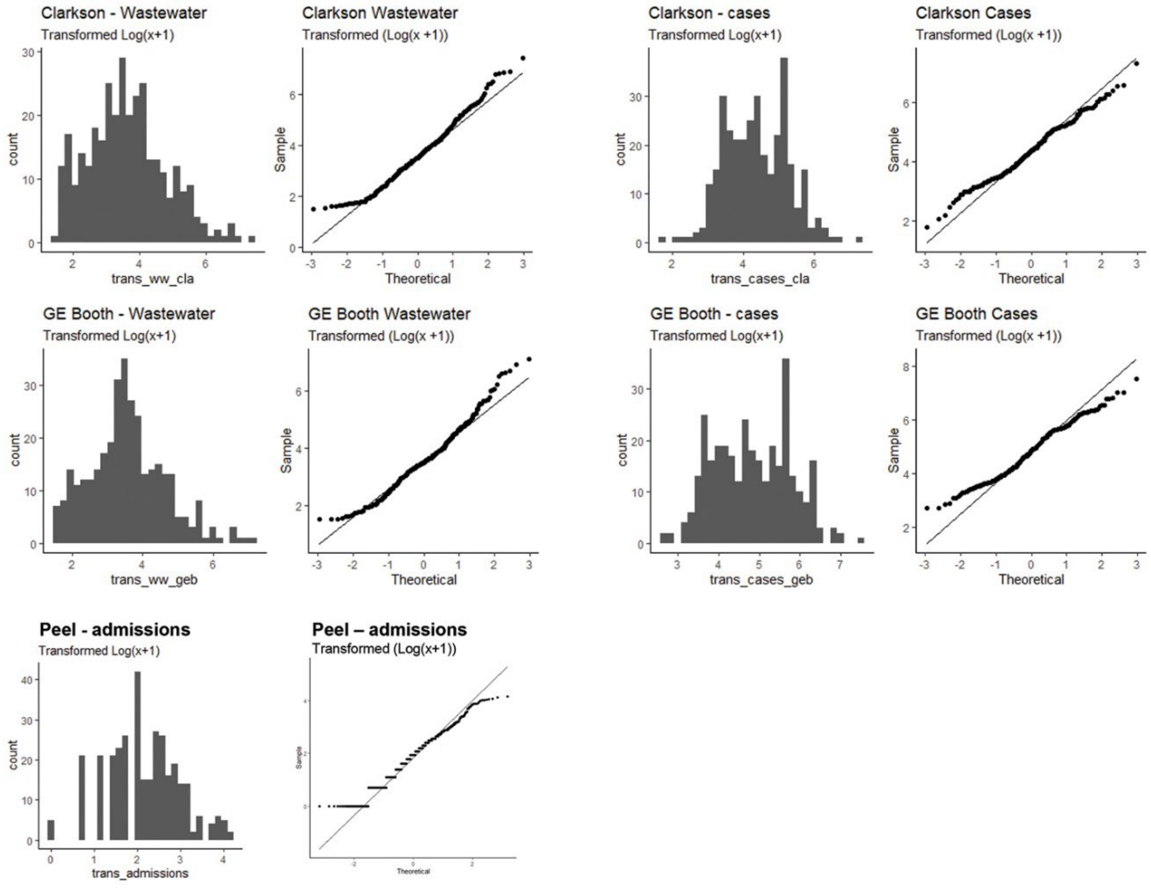

Appendix Figure 1. Histograms and quantile-quantile plots of natural ln(x+1) transformed wastewater SARS-CoV-2 concentration data and COVID-19 case and hospitalization data.

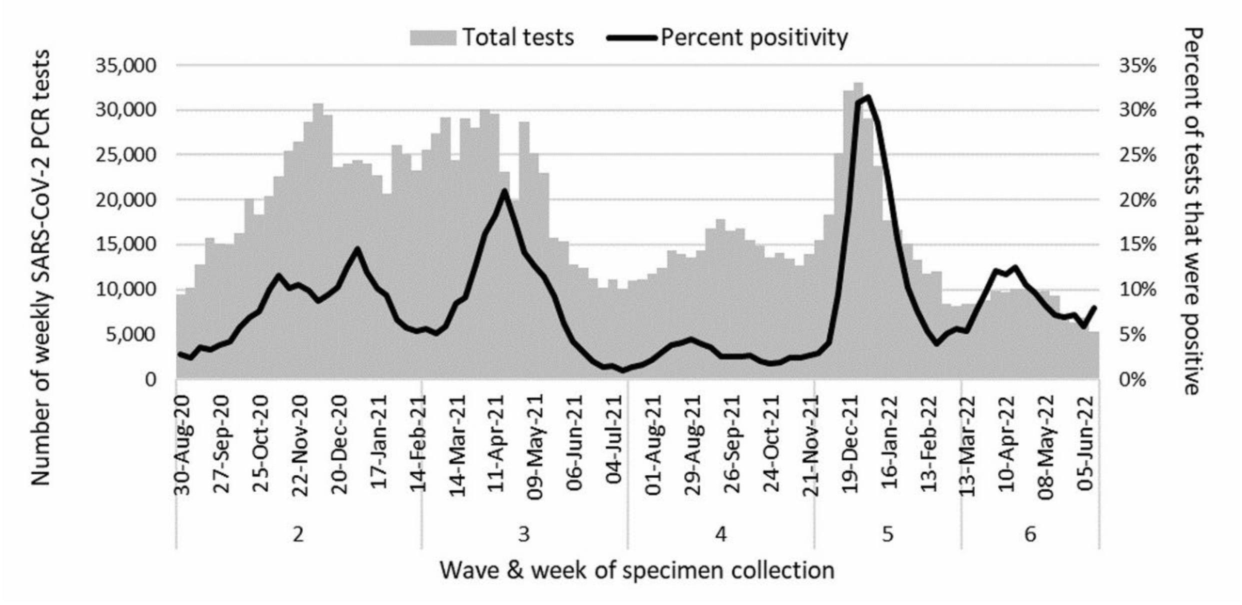

Appendix Figure 2. Weekly number of SARS-CoV-2 clinical PCR tests and percent positivity, August 30, 2020 to June 18, 2022, Peel region.

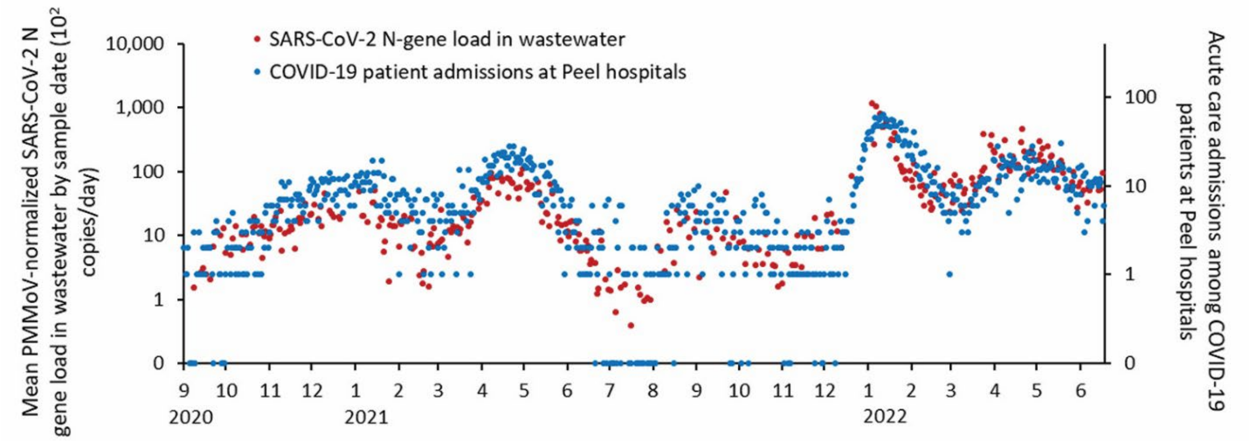

Appendix Figure 3. Combined PMMoV-normalized SARS-CoV-2 N-gene load in untreated wastewater at Clarkson and G.E. Booth Wastewater Treatment Plants and acute admissions of confirmed COVID-19 patients at Peel hospitals, September 1, 2020 to June 18, 2022. Data are plotted on the logarithmic scale. For data visualization purposes, daily hospitalization values of zero were converted to 0.1 and are shown along the x-axis.

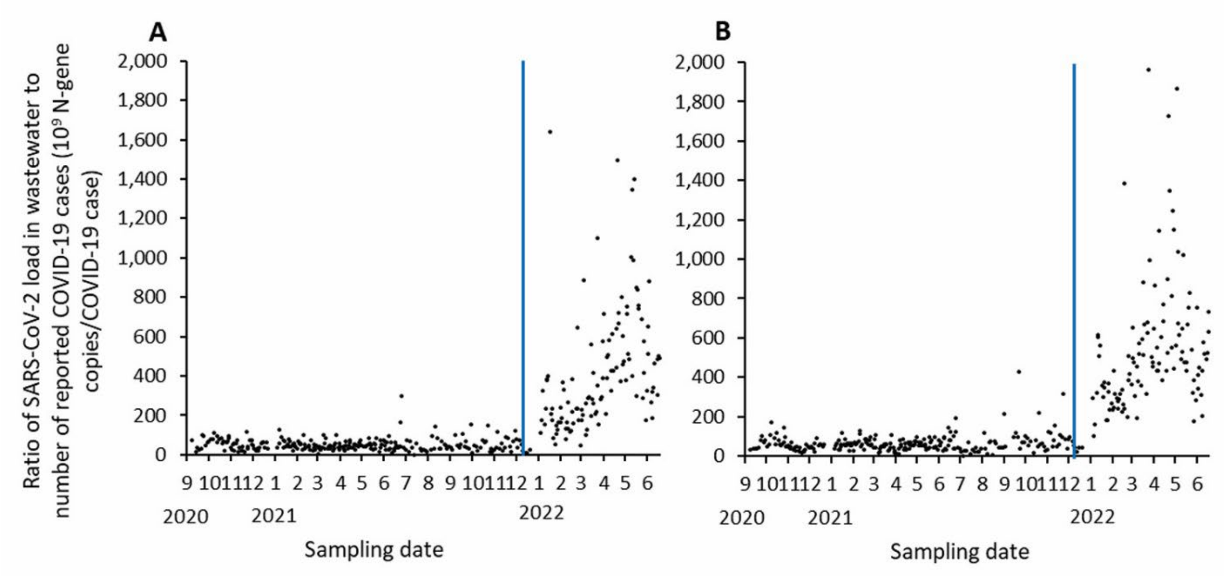

Appendix Figure 4. Ratio of daily SARS-CoV-2 N-gene load in wastewater to concurrent reported Peel COVID-19 cases by WWTP, September 1, 2020 to June 18, 2022. A) Clarkson WWTP, B) G.E. Booth WWTP. The blue line marks the date when clinical PCR testing for SARS-CoV-2 was restricted to high-risk populations, December 31, 2021.
